# The association of antiviral drugs with COVID-19 morbidity: The retrospective analysis of a nationwide COVID-19 cohort

**DOI:** 10.3389/fmed.2022.894126

**Published:** 2022-08-31

**Authors:** Cenk Babayigit, Nurdan Kokturk, Seval Kul, Pelin Duru Cetinkaya, Sibel Atis Nayci, Serap Argun Baris, Oguz Karcioglu, Pinar Aysert, Ilim Irmak, Aycan Akbas Yuksel, Yonca Sekibag, Oya Baydar Toprak, Emel Azak, Sait Mulamahmutoglu, Caglar Cuhadaroglu, Aslihan Demirel, Bugra Kerget, Burcu Baran Ketencioglu, Hasan Selcuk Ozger, Gulcihan Ozkan, Zeynep Ture, Begum Ergan, Vildan Avkan Oguz, Oguz Kilinc, Merve Ercelik, Tansu Ulukavak Ciftci, Ozlem Alici, Esra Nurlu Temel, Ozlem Ataoglu, Asena Aydin, Dilek Cetiner Bahcetepe, Yusuf Taha Gullu, Fusun Fakili, Figen Deveci, Neslihan Kose, Muge Meltem Tor, Gulsah Gunluoglu, Sedat Altin, Teyfik Turgut, Tibel Tuna, Onder Ozturk, Oner Dikensoy, Pinar Yildiz Gulhan, Ilknur Basyigit, Hasim Boyaci, Ipek Kivilcim Oguzulgen, Sermin Borekci, Bilun Gemicioglu, Firat Bayraktar, Osman Elbek, Ismail Hanta, Hacer Kuzu Okur, Gulseren Sagcan, Oguz Uzun, Metin Akgun, Goksel Altinisik, Berna Dursun, Ebru Cakir Edis, Erkmen Gulhan, Fusun Oner Eyuboglu, Okkes Gultekin, Yavuz Havlucu, Metin Ozkan, Aysin Sakar Coskun, Abdullah Sayiner, A. Fuat Kalyoncu, Oya Itil, Hasan Bayram

**Affiliations:** ^1^Department of Pulmonary Medicine, Faculty of Medicine, Mustafa Kemal University, Antakya, Turkey; ^2^Department of Pulmonary Medicine, Faculty of Medicine, Gazi University, Ankara, Turkey; ^3^Department of Biostatistics, Faculty of Medicine, Gaziantep University, Gaziantep, Turkey; ^4^Department of Pulmonary Medicine, Adana City Training and Research Hospital, University of Health Sciences, Adana, Turkey; ^5^Department of Pulmonary Medicine, Faculty of Medicine, Çukurova University, Adana, Turkey; ^6^Department of Pulmonary Medicine, Faculty of Medicine, Mersin University, Yenişehir, Turkey; ^7^Department of Pulmonary Medicine, Faculty of Medicine, Kocaeli University, İzmit, Turkey; ^8^Department of Pulmonary Medicine, Halil Şıvgın Cubuk State Hospital, Ankara, Turkey; ^9^Department of Infectious Disease, Faculty of Medicine, Gazi University, Ankara, Turkey; ^10^Department of Pulmonary Medicine, Faculty of Medicine, Hacettepe University, Ankara, Turkey; ^11^Department of Pulmonary Medicine, Faculty of Medicine, Ufuk University, Ankara, Turkey; ^12^Department of Pulmonary Disease, Cerrahpaşa Faculty of Medicine, Istanbul University-Cerrahpasa, Istanbul, Turkey; ^13^Department of Infectious Disease and Clinical Microbiology, Faculty of Medicine, Kocaeli University, İzmit, Turkey; ^14^Department of Pulmonary Medicine, Faculty of Medicine, Altunizade Acibadem Hospital, Acibadem University, Istanbul, Turkey; ^15^Department of Infectious Disease, Kadıköy Florence Nightingale Hospital, Istanbul, Turkey; ^16^Department of Pulmonary Medicine, Faculty of Medicine, Ataturk University, Erzurum, Turkey; ^17^Department of Pulmonary Medicine, Faculty of Medicine, Erciyes University, Kayseri, Turkey; ^18^Department of Pulmonary Medicine, Acibadem Maslak Hospital, Istanbul, Turkey; ^19^Operating Room Services Department, Vocational School, Nişantaşı University, Istanbul, Turkey; ^20^Department of Infectious Disease and Clinical Microbiology, Faculty of Medicine, Erciyes University, Kayseri, Turkey; ^21^Department of Pulmonary Medicine, Faculty of Medicine, Dokuz Eylul University, Izmir, Turkey; ^22^Department of Infectious Disease and Clinical Microbiology, Faculty of Medicine, Dokuz Eylul University, Izmir, Turkey; ^23^Department of Pulmonary Medicine, Faculty of Medicine, Düzce University, Düzce, Turkey; ^24^Department of Infectious Disease, Turkiye Gazetesi Private Hospital, Istanbul, Turkey; ^25^Department of Infectious Diseases and Clinical Microbiology, Faculty of Medicine, Suleyman Demirel University, Isparta, Turkey; ^26^Department of Pulmonary Medicine, Kestel State Hospital, Bursa, Turkey; ^27^Department of Pulmonary Medicine, Faculty of Medicine, Ondokuz Mayıs University, Samsun, Turkey; ^28^Department of Pulmonary Medicine, Faculty of Medicine, Gaziantep University, Gaziantep, Turkey; ^29^Department of Pulmonary Medicine, Faculty of Medicine, Firat University, Elazı$\overset{˘}{\text{g}}$, Turkey; ^30^Department of Pulmonary Medicine, Bilecik Training and Research Hospital, Bilecik, Turkey; ^31^Department of Pulmonary Medicine, Faculty of Medicine, Zonguldak Bülent Ecevit University, Zonguldak, Turkey; ^32^Department of Pulmonary Medicine, Yedikule Chest Diseases and Chest Surgery Training and Research Hospital, University of Health Science, Istanbul, Turkey; ^33^Department of Pulmonary Medicine, Faculty of Medicine, Suleyman Demirel University, Isparta, Turkey; ^34^Department of Pulmonary Medicine, Faculty of Medicine, Taksim, Acibadem University, Istanbul, Turkey; ^35^Department of Internal Medicine, Faculty of Medicine, Dokuz Eylul University, Izmir, Turkey; ^36^Department of Pulmonary Medicine, Kadıköy Florence Nightingale Hospital, Istanbul, Turkey; ^37^Department of Pulmonary Medicine, Faculty of Medicine, Pamukkale University, Denizli, Turkey; ^38^Department of Pulmonary Medicine, Ankara Memorial Hospital, Ankara, Turkey; ^39^Department of Pulmonary Medicine, Faculty of Medicine, Trakya University, Edirne, Turkey; ^40^Department of Thoracic Surgery, Atatürk Chest Diseases and Thoracic Surgery Training and Research Hospital, Ankara, Turkey; ^41^Department of Pulmonary Medicine, School of Medicine, Başkent University, Ankara, Turkey; ^42^Department of Pulmonary Medicine, Faculty of Medicine, Celal Bayar University, Manisa, Turkey; ^43^Department of Pulmonary Medicine, Faculty of Medicine, Ege University, Izmir, Turkey; ^44^Department of Pulmonary Medicine, Koç University School of Medicine, Istanbul, Turkey; ^45^Koç University Research Center for Translational Medicine (KUTTAM), Koç University School of Medicine, Istanbul, Turkey

**Keywords:** antiviral agents, COVID-19 morbidity, length of hospitalization, ICU requirement, invasive mechanical ventilation

## Abstract

**Background and objectives:**

Although several repurposed antiviral drugs have been used for the treatment of COVID-19, only a few such as remdesivir and molnupiravir have shown promising effects. The objectives of our study were to investigate the association of repurposed antiviral drugs with COVID-19 morbidity.

**Methods:**

Patients admitted to 26 different hospitals located in 16 different provinces between March 11–July 18, 2020, were enrolled. Case definition was based on WHO criteria. Patients were managed according to the guidelines by Scientific Board of Ministry of Health of Turkey. Primary outcomes were length of hospitalization, intensive care unit (ICU) requirement, and intubation.

**Results:**

We retrospectively evaluated 1,472 COVID-19 adult patients; 57.1% were men (mean age = 51.9 ± 17.7years). A total of 210 (14.3%) had severe pneumonia, 115 (7.8%) were admitted to ICUs, and 69 (4.7%) were intubated during hospitalization. The median (interquartile range) of duration of hospitalization, including ICU admission, was 7 (5–12) days. Favipiravir (*n* = 328), lopinavir/ritonavir (*n* = 55), and oseltamivir (*n* = 761) were administered as antiviral agents, and hydroxychloroquine (HCQ, *n* = 1,382) and azithromycin (*n* = 738) were used for their immunomodulatory activity. Lopinavir/ritonavir (β [95% CI]: 4.71 [2.31–7.11]; *p* = 0.001), favipiravir (β [95% CI]: 3.55 [2.56–4.55]; *p* = 0.001) and HCQ (β [95% CI]: 0.84 [0.02–1.67]; *p* = 0.046) were associated with increased risk of lengthy hospital stays. Furthermore, favipiravir was associated with increased risks of ICU admission (OR [95% CI]: 3.02 [1.70–5.35]; *p* = 0.001) and invasive mechanical ventilation requirement (OR [95% CI]: 2.94 [1.28–6.75]; *p* = 0.011).

**Conclusion:**

Our findings demonstrated that antiviral drugs including lopinavir, ritonavir, and favipiravir were associated with negative clinical outcomes such as increased risks for lengthy hospital stay, ICU admission, and invasive mechanical ventilation requirement. Therefore, repurposing such agents without proven clinical evidence might not be the best approach for COVID-19 treatment.

## Introduction

Severe acute respiratory syndrome coronavirus 2 (SARS-CoV-2) pandemic started in Wuhan, China, at the end of 2019, and spread rapidly to many countries. The disease was termed COVID-19 by March 11, 2020. Globally, as of June 30, 2022, there have been more than 543 million confirmed cases of COVID-19, including 6.33 million deaths reported to the World Health Organization (WHO) (1).

**FIGURE 1:**
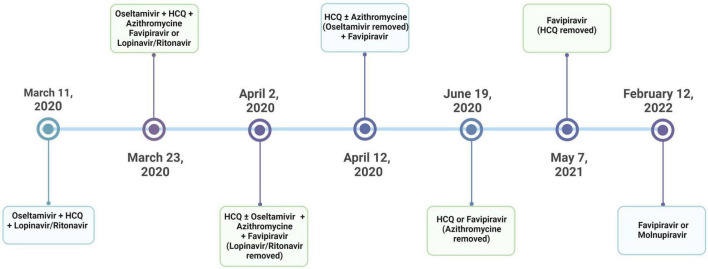
The timeline for the treatment protocols applied by the guidelines by the Scientific Board of the Ministry of Health (SBMH) of Turkey.

Several repurposed antiviral agents have been administered in the treatment of COVID-19 worldwide (2–9). Although there are many ongoing studies, only a few drugs such as remdesivir and molnupiravir, which are not available in many countries, have shown promising effects. In Turkey, the treatment guidelines for adult patients with COVID-19 have been prepared and regularly updated by the Scientific Board of the Ministry of Health (SBMH), since March 2020 (10). While hydroxychloroquine (HCQ) was recommended for mild cases and lopinavir/ritonavir combination for moderate and severe patients with COVID-19 in the first version of this guideline, favipiravir was implemented as a new recommendation for progressive mild, moderate or severe cases in the second version, which was published on March 23, 2020. On April 2, 2020, the lopinavir/ritonavir combination was removed from moderate and severe cases and recommended only for pregnant patients (Figure 1). Eventually, favipiravir was widely accepted and used by pulmonologists for moderate and severe COVID-19 cases in Turkey.

Real-world data about the effectiveness of antiviral agents, including favipiravir, is limited. TTD-TURCOVID-19 is a registry that contains data from 26 centers in 16 different provinces and thus may be considered representative of Turkey. In this *post hoc* study, we aimed to investigate the association between antiviral agents, HCQ, and azithromycin and the morbidity of patients of COVID-19 using data from the TTD-TURCOVID-19 registry. Mortality data obtained in this registry have been previously published elsewhere (11).

## Materials and methods

### Study design

The study was approved by the Institutional Review Board of Gazi University Faculty of Medicine, Ankara, Turkey (356/22.05.2020), and partially supported by the Turkish Thoracic Society (TTS). The study analyzed retrospectively collected data from the hospital records to evaluate the clinical outcomes of hospitalized patients. No informed consent was required, because of the retrospective design of the study, and a waiver of informed consent was obtained for the study from the same institutional review board. All human studies conformed to the ethical principles for medical research involving human subjects, as set out in the World Medical Association Declaration of Helsinki. Patients admitted to 26 different hospitals (17 university hospitals, 2 large tertiary hospitals, 2 secondary care hospitals, and 5 private hospitals) located in 16 different provinces between March 11 and July 18, 2020, were consecutively enrolled, and the details of the study design were published previously (11). Briefly, the inclusion criteria were; (i) adult patients (age of ≥ 16 years) with a diagnosis of COVID 19 according to WHO criteria; a definite diagnosis (proven with a positive PCR test) or probable COVID-19 pneumonia based on a typical clinical presentation following contact with a patient who had a definite diagnosis, together with typical CT findings (predominantly peripheral ground glass opacities with or without areas of consolidation) but not confirmed with a PCR test (12), (ii) patients, who were treated with any of drugs including lopinavir, ritonavir, favipiravir, HCQ, or azithromycin. Patients who did not take any of treatment drugs, and those with incomplete records were excluded. All centers participated in the registry voluntarily following the call by the TTS for TTD-TURCOVID-19 registry.

The final diagnoses were made according to previously published guidelines (12–16). Accordingly, patients were diagnosed with a spectrum of asymptomatic, mild to moderate acute respiratory diseases, including non-severe pneumonia and severe/critical diseases, such as severe pneumonia, adult respiratory distress syndrome (ARDS), multiple organ dysfunction syndrome (MODS), sepsis or septic shock. More than one of these conditions could be registered in the database.

All patients were managed according to the SBMH treatment guidelines (10). Accordingly, patients received HCQ and/or lopinavir/ritonavir, whereas favipiravir was recommended for moderate or severe cases. If a mild case was unresponsive to the initial treatment with HCQ, either lopinavir/ritonavir or favipiravir was added to HCQ. In addition, oseltamivir was recommended for cases in which influenza could not be excluded. Thus, while oseltamivir took part in the early versions of the treatment guidelines regardless of the disease severity, it was removed from the updated guidelines published on April 12, 2020. Similarly, azithromycin was implemented according to physicians’ decision for possible or definitive COVID-19 cases with pneumonia; however, it was excluded from the guidelines in June 2020 (Figure 1).

The clinical data, comorbidities, final diagnoses, laboratory findings, drugs used in the treatment, adverse events, and complications were noted from hospital records. Prolonged QTc was defined as an increase of more than 60 ms (ΔQTc > 60 ms) in QTc intervals compared to pre-treatment ECG or a QTc of 500 ms or above (17). Acute hepatotoxicity and acute renal toxicity were noted by attending physicians based on hepatic test (alanine aminotransferase, ALT or aspartate aminotransferase, AST) abnormalities and renal function test (a reduction in glomerular filtration rate) abnormalities, respectively. The data were recorded in an internet-based database by attending physicians and were rechecked with the source documents for accuracy prior to the statistical analysis.

Primary outcomes of the study were the length of hospitalization, the requirement of an intensive care unit (ICU) and intubation during hospitalization. The secondary outcomes were related to QT prolongation in the electrocardiogram (ECG) and liver and renal function test abnormalities.

### Statistical analysis

First, univariate analyses were performed to evaluate the association of treatments with morbidity outcomes. The Mann–Whitney *U*-test (for continuous variables) and chi-squared test (for categorical variables) were used, and odds ratios (ORs) and 95% confidence intervals (CIs) were calculated for categorical outcomes. Second, multivariate binary logistic regression models for categorical outcomes and generalized linear regression models for numerical outcomes were built to adjust for the effect of potential confounding factors on morbidities. Adjusted beta coefficients (β) and ORs were given to show the effect size in numerical and categorical variables, respectively. Clinically related variables were included in the model if significant at the 10% level according to the univariate analysis results. Multicollinearity was checked by calculating variance inflation factors. All univariate analyses were performed in SPSS for Windows version 22.0, and a two-sided *P*-value < 0.05 was defined as statistically significant.

## Results

**FIGURE 2:**
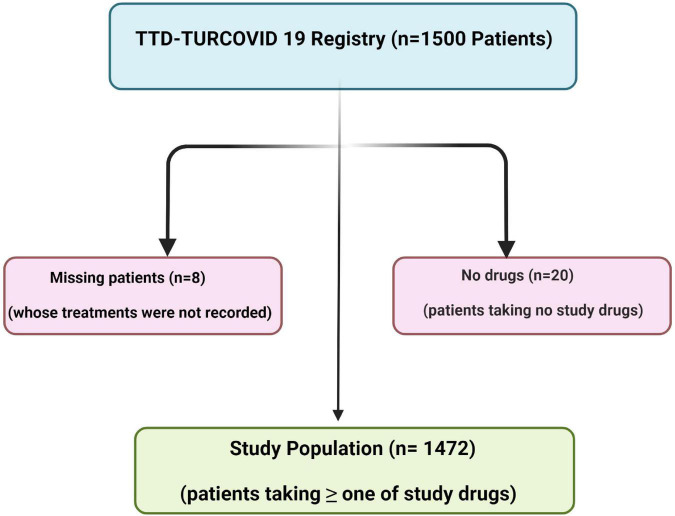
The enrollment of the study population.

A total of 1,500 patients were recruited from the TTD-TURCOVID-19 registry (11). However, the records of treatment data of 8 patients were missing, and 20 patients did not take repurposed drugs for their treatment. Accordingly, the final number of our study population was 1,472 (Figure 2). Of these, the diagnosis was confirmed with PCR in 1,036 (70.4%), and 436 patients (29.6%) had highly probable COVID-19. Of the patients, 57.1% male and 25.4% were ≥ 65 years old (mean age ± *SD* = 51.9 ± 17.7 years). The median (interquartile range, IQR) for number of comorbidities was 0 (0–1). Of all cases, 1,129 (76.7%) were diagnosed with non-severe pneumonia, whereas 210 (14.3%) had severe pneumonia. A total of 115 (7.8%) were admitted to an ICU, and 69 (4.7%) were intubated during hospitalization. The data on the initial treatment setting were recorded for 1,317 patients. Most of these patients 1,161 (78.9%) were hospitalized in wards, 46 (3.1%) were admitted directly to the ICU, and 110 (7.5%) were treated as outpatients, whereas the initial treatment setting was not recorded in 155 patients (10.5%). The median (IQR) of duration of hospitalization including ICU stay, was 7 (5–12) days. Demographic characteristics, initial treatment setting, and final spectrum of the disease are summarized in Table 1.

**TABLE 1:** Demographic characteristics, initial treatment setting, and disease spectrum.

	*n*	%
Study population:	1,472	100
Confirmed cases	1,036	70.4
Highly probable cases	436	29.6
Male	836	57.1
Female	627	42.9
Patients < 65 years old	1,096	74.6
≥ 65 years old	373	25.4
**Initial treatment setting (recorded)**		
Community	110	7.5
Non-ICU hospitalization	1,161	78.9
ICU	46	3.1
Missing data	155	10.5
Final spectrum of the disease*		
Asymptomatic	117	7.9
Acute lower respiratory disease	30	2.5
Pneumonia	1,129	76.7
Severe pneumonia	210	14.3
ARDS	34	2.3
MODS	18	1.2
Sepsis	33	2.2
Septic shock	8	0.5
MAS	21	1.4
Others^#^	3	0.2

*The attending physician could choose more than one diagnosis. ^#^Patients with any other symptoms/diagnosis. ICU, intensive care unit. ARDS, adult respiratory distress syndrome. MAS, macrophage activation syndrome. MODS, multiorgan dysfunction syndrome.

### Pharmacological therapy

**FIGURE 3:**
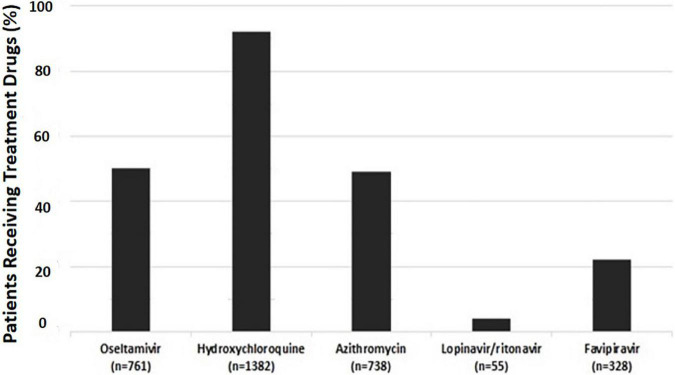
Frequency of the patients, who were administered any of the given drugs; either alone or in combination with other drugs.

Figure 3 shows the frequency of drugs given to the patients, either alone or in combination with other drugs. HCQ was the most frequently administered drug in the study population. Favipiravir was used in 328 (25.1%) patients. Of these, 307 were taking a combination of medications that included other antiviral or immunomodulatory drugs. Supplementary Figure 1 demonstrates distribution of patients with different drug combinations. A total of 396 patients received antibiotics, other than azithromycin. Overall, the median (IQR) of antibiotics consumed was 0 (0–1).

### Association of drugs with morbidity

The association of antiviral drugs with morbidity parameters such as the length of hospital stay, ICU need, and the requirement of invasive mechanical ventilation (IMV) were evaluated by univariate and generalized linear regression analyses (Table 2), and by univariate and multivariate binary logistic regression analyses (Tables 3, 4). There was a positive association between an increased risk of lengthy hospital stay and treatment with lopinavir/ritonavir (β [95% CI]: 4.71 [2.31–7.11]; *p* = 0.001), favipiravir (β [95% CI]: 3.55 [2.56–4.55]; *p* = 0.001), and HCQ (β [95% CI]: 0.84 [0.02–1.67]; *p* = 0.046), respectively. Similarly, favipiravir usage was associated with an increased risk of required symptoms for admittance to the ICU (OR [95% CI]: 3.02 [1.70–5.35]; *p* = 0.001) and IMV (OR [95% CI]: 2.94 [1.28–6.75]; *p* = 0.011).

**TABLE 2:** The association between use of treatment drugs and the duration of hospital stay.

	Length of hospital stay Univariate analysis			Generalized linear regression analyses*	
Treatments	*n*	Median (25–75%)	*P*	β (95% CI)	*P*
Oseltamivir			0.002		0.173
Yes	586	7 (5–12)		0.56 (−1.36–1.86)	
No	476	7 (5–10)		1 (reference)	
Lopinavir/ritonavir			0.001		0.001*
Yes	41	14 (8–17)		4.71 (2.31–7.11)	
No	928	7 (5–10)		1 (reference)	
Favipiravir			0.001		0.001*
Yes	265	12 (8–17)		3.55 (2.56–4.55)	
No	746	6 (5–9)		1 (reference)	
Hydroxychloroquine			0.052		0.046*
Yes	852	7 (5–12)		0.84 (0.02–1.67)	
No	63	6 (4–10)		1 (reference)	
Azithromycin			0.009		0.081
Yes	505	7 (5–12)		1.39 (−0.17–2.97)	
No	410	6 (5–10)		1 (reference)	

*For antivirals; adjusted by number of comorbidities, number of antibiotics used, age, sex, severe-critical disease, hydroxychloroquine use, azithromycin use, and for hydroxychloroquine and azithromycin; adjusted by number of comorbidities, number of antibiotics used, age, sex severe-critical disease and any antiviral use.

β, regression coefficient.

**TABLE 3:** The association of treatment drugs with the requirement of ICUs.

		ICU requirement		Univariate analysis		Multivariate binary logistic regression analysis*	
			
		Yes *N* (%)	No *N* (%)	OR (95% CI)	*P*	OR (95% CI)	*P*
Oseltamivir	Yes	69 (60)	681 (54.4)	1.26 (0.85–1.85)	0.252	0.77 (0.44–1.36)	0.774
	No	46 (40)	570 (45.6)	1 (reference)		1 (reference)	
Lopinavir/Ritonavir	Yes	14 (16.1)	41 (3.6)	5.16 (2.69–9.89)	0.001	1.60 (0.57–4.51)	0.372
	No	73 (83.9)	1,103 (96.4)	1 (reference)		1 (reference)	
Favipiravir	Yes	75 (68.2)	250 (21.1)	8.01 (5.24–12.24)	0.001	3.02 (1.70–5.35)	0.001*
	No	35 (31.8)	934 (78.9)	1 (reference)		1 (reference)	
Hydroxychloroquine	Yes	107 (92.2)	1,256 (93.7)	0.79 (0.39–1.63)	0.530	1.62 (0.61–4.3)	0.330
	No	9 (7.8)	84 (6.3)	1 (reference)		1 (reference)	
Azithromycin	Yes	61 (57.5)	673 (54.7)	1.12 (0.75–1.67)	0.574	1.25 (0.69–2.25)	0.465
	No	45 (42.5)	557 (45.3)	1 (reference)		1 (reference)	

*For antivirals; adjusted for number of comorbidities, number of antibiotics used, age, sex severe-critical disease, hydroxychloroquine use, azithromycin use, and for hydroxychloroquine and azithromycin adjusted for number of comorbidities, number of antibiotics used, age, sex severe-critical disease and any antiviral use.

OR, Odds ratio, CI: Confidence Interval.

**TABLE 4:** The association between the use of treatment drugs and the requirement for invasive mechanical ventilation.

	Invasive mechanical ventilation requirement		Univariate analyses		Multi variate binary logistic regression analysis	
			
	Yes *n* (%)	No *n* (%)	OR (95%CI)	*P*	OR (95%CI)	*P*
**Oseltamivir**						
Yes No	44 (63.8)	713 (54.3)	1.48 (0.89–2.44)	0.127	1.05 (0.47–2.35)	0.898
	25 (36.2)	599 (45.7)	1 (reference)		1 (reference)	
**Lopinavir/ritonavir**						
Yes	12 (24.5)	43 (3.6)	8.65	0.001	2.01 (0.56–6.94)	0.270
No	37 (75.5)	1,147 (96.4)	(4.22–17.51)		1 (reference)	
**Favipiravir**						
Yes	47 (73.4)	278 (22.5)	9.55 (5.39–16.89)	0.001	2.94 (1.28–6.75)	0.011*
No	17 (26.6)	960 (77.5)	1 (reference)		1 (reference)	
**Hydroxychloroquine**						
Yes	63 (91.3)	1,315 (93.7)	1.42 (0.60–3.38)	0.424	2.3 (0.63–8.4)	0.209
No	6 (8.7)	88 (6.3)	1 (reference)		1 (reference)	
**Azithromycin**						
Yes	33 (55)	701 (54.6)	1.02 (0.61–1.71)	0.946	1.56 (0.68–3.62)	0.297
No	27 (45)	584 (45.4)	1 (reference)		1 (reference)	

*For antivirals, adjusted for number of comorbidities, number of antibiotics used, age, sex severe-critical disease, hydroxychloroquine use, azithromycin use, and for hydroxychloroquine and azithromycin adjusted for number of comorbidities, number of antibiotics used, age, sex severe-critical disease and any antiviral use.

OR, Odds ratio; CI, Confidence Interval.

### Side effects of antiviral drugs

QTc prolongation was present in 37 (3%) of patients. The univariate and subsequent multivariate binary logistic regression analyses with multiple adjustments are shown in Table 5.

**TABLE 5:** QTc prolongation during treatment with drugs.

	QTc prolongation		Univariate analysis		Multivariate binary logistic regression analysis*	
			
	Yes	No	OR (95%CI)	*P*	OR (95%CI)	*P*
**Oseltamivir**						
Yes	18 (48.6)	627 (56.1)	0.74 (0.39–1.43)	0.372	0.49 (0.23–1.076)	0.076
No	19 (51.4)	491 (43.9)	1 (reference)		1 (reference)	
**Lopinavir/ritonavir**						
Yes	3 (9.7)	47 (4.7)	2.18 (0.64–7.44)	0.212	1.23 (0.16–9.65)	0.842
No	28 (90.3)	957 (95.3)	1 (reference)		1 (reference)	
**Favipiravir**						
Yes	15 (44.1)	293 (27.6)	2.07 (1.04–4.13)	0.039	1.75 (0.75–4.07)	0.195
No	19 (55.9)	769 (72.4)	1 (reference)		1 (reference)	
**Hydroxychloroquine**						
Yes	36 (97.3)	1,139 (95.7)	1.61 (0.22–11.99)	0.641	1.51 (0.19–11.75)	0.687
No	1 (2.7)	51 (4.3)	1 (reference)		1 (reference)	
**Azithromycin**						
Yes	25 (67.6)	622 (57.1)	1.56 (0.78–3.15)	0.210	1.3 (0.59–2.87)	0.515
No	12 (32.4)	467 (42.9)	1 (reference)		1 (reference)	

*For Antivirals; adjusted by hydroxychloroquine, azithromycin, number of antibiotics used, hypertension and heart failure, and for hydroxychloroquine and azithromycin; adjusted by number of antibiotics used, hypertension, heart failure and any antiviral use.

Acute hepatic and renal toxicity were present in 79 (6%) and 32 (2.4%) of the patients, respectively. While lopinavir/ritonavir was associated with increased risks both of hepatic (OR [95% CI]: 5.41 [2.30–12.68]; *p* = 0.001) and renal toxicity (OR [95% CI]: 5.07 [1.29–19.85]; *p* = 0.02), respectively, there was an association between favipiravir and increased risk of only hepatic toxicity (OR [95% CI]: 3.20 [1.88–5.46]; *p* = 0.001). Similarly, azithromycin was associated with an increase in the risk for hepatotoxicity (OR [95% CI]: 2.31 [1.34–2.39]; *p* = 0.003) (Table 6).

**TABLE 6:** Acute hepatic toxicity and acute renal toxicity during treatment with drugs.

	Acute hepatic toxicity		Univariate analysis		Multivariate binary logistic regression analysis*	
			
	Yes	No	OR (95%CI)	*P*	OR (95%CI)	*P*
**Oseltamivir**						
Yes	41 (52.6)	657 (53.6)	0.96 (0.61–1.52)	0.854	0.80 (0.49–1.32)	0.387
No	37 (47.4)	568 (46.4)	1 (reference)		1 (reference)	
**Lopinavir/Ritonavir**						
Yes	11 (14.3)	41 (3.6)	4.48 (2.2–9.12)	0.001	5.41 (2.30–12.68)	0.001*
No	66 (85.7)	1,103 (96.4)	1 (reference)		1 (reference)	
**Favipiravir**						
Yes	42 (53.2)	261 (22.1)	4.01 (2.52–6.36)	0.001	3.20 (1.88–5.46)	0.001*
No	37 (46.8)	921 (77.9)	1 (reference)		1 (reference)	
**Hydroxychloroquine**						
Yes	78 (98.7)	1,145 (92.7)	6.13 (0.84–44.59)	0.073	5.12 (0.69–38.18)	0.111
No	1 (1.3)	90 (7.3)	1 (reference)		1 (reference)	
**Azithromycin**						
Yes	50 (65.8)	615 (51.6)	1.8 (1.11–2.93)	0.018	2.31 (1.34–2.39)	0.003*
No	26 (34.2)	576 (48.4)	1 (reference)		1 (reference)	

	**Acute renal toxicity**		**Univariate analysis**			
			
	**Yes**	**No**	**OR [95%CI]**	***P***	**OR [95%CI]**	***P***

**Oseltamivir**						
Yes	20 (64.5)	678 (53.3)	1.59 (0.76–3.35)	0.220	1.47 (0.61–3.51)	0.393
No	11 (35.5)	594 (46.7)	1 (reference)		1 (reference)	
**Lopinavir/Ritonavir**						
Yes	5 (16.1)	47 (3.9)	4.68 (1.72–12.72)	0.003	5.07 (1.29–19.85)	0.020*
No	26 (83.9)	1,143 (96.1)	1 (reference)		1 (reference)	
**Favipiravir**						
Yes	16 (50)	287 (23.4)	3.28 (1.62–6.65)	0.001	1.75 (0.70–4.36)	0.231
No	16 (50)	942 (76.6)	1 (reference)		1 (reference)	
**Hydroxychloroquine**						
Yes	30 (93,9)	1,193 (93.1)	1.12 (0.26–4.76)	0.879	1.05 (0.22–4.92)	0.956
No	2 (6,3)	89 (6.9)	1 (reference)		1 (reference)	
**Azithromycin**						
Yes	17 (58.6)	648 (52.3)	1.29 (0.61–2.72)	0.504	1.64 (0.67–4.02)	0.278
No	12 (41.4)	590 (47.7)	1 (reference)		1 (reference)	

*For antivirals; adjusted by hydroxychloroquine, azithromycin, number of comorbidities and antibiotics, and for hydroxychloroquine and azithromycin; adjusted by number of comorbidities, number of antibiotics, and any antiviral use.

## Discussion

In the current study, we investigated the association of antiviral drugs including favipiravir, oseltamivir, and lopinavir/ritonavir, and HCQ and azithromycin with morbidity measures such as length of hospital stay, admission to the ICU and IMV. None of the drugs showed an association with the improvement of clinical outcomes. In contrast, favipiravir, lopinavir/ritonavir and HCQ were associated with longer hospitalization. Furthermore, favipiravir was significantly associated with increased the risk of ICU admission and the requirement for IMV. Eventually, these findings suggest that favipiravir, lopinavir/ritonavir and HCQ can worsen the clinical outcomes of COVID-19 patients.

COVID-19 pandemic has challenged the physicians, scientists, and health care providers worldwide with both the increased number of patients and its mortal effects. This led to both physicians, the scientific community, and decision makers to consider repurposing the existing antiviral drugs, immunomodulators, even antibiotics in the treatment of the disease (2–9).

HCQ and azithromycin were used widely as immunomodulators worldwide at the beginning of the pandemic. Several *in vitro* studies and clinical trials showed that chloroquine had a significant effect on both clinical outcomes and viral clearance of SARS-CoV-2 (18, 19). Consequently, HCQ became a part of the standard regimen in the treatment of COVID-19 in China and many countries and states followed, such as Spain, Iran, Turkey and New York, United States (20). However, subsequent studies did not show clinical benefits from these drugs; therefore, the WHO and several other medical organizations opposed its use except in clinical trials (21–25). The U.S. Food and Drug Administration (FDA) revoked emergency use authorization for chloroquine and HCQ on June 15, 2020, and following results from its Interim Solidarity Trial, WHO discontinued HCQ and lopinavir/ritonavir treatment arms for COVID-19 on July 4, 2020 (21, 22).

In the light of these actions, HCQ was removed from treatment recommendations for COVID-19 in many countries. However, although azithromycin was removed from recommendations in June 2020, HCQ remained in SBMH guidelines until May 2021 (10). As a result, most of our study population used HCQ and azithromycin. When we analyzed our data on the clinical outcomes such as hospital stay, ICU admission, and IMV requirement, neither of these drugs showed a clinical benefit. In contrast, HCQ was associated with an increase in the risk of longer hospital stay.

An anti-influenza drug, oseltamivir, was also recommended by SBMH as an initial therapy for cases in which influenza was confirmed or could not be excluded because the time of the pandemic overlapped with the flu season in the Northern Hemisphere (10). Therefore, approximately half of the patients involved in this study received oseltamivir during the first wave of the pandemic. Unadjusted analysis revealed that oseltamivir was associated with longer hospital stays, and this association reverted with multiple adjustments.

Although the lopinavir/ritonavir combination was originally used for the treatment of acquired immunodeficiency syndrome, it was also included in a standard protocol as an initial treatment for SARS-CoV-1, since initial studies reported their clinical benefits (26–28). After an *in vitro* study showing the antiviral activity of lopinavir against SARS-CoV-2, the combination of the two drugs was implemented in the treatment of COVID-19 (29). However, several clinical trials failed to demonstrate the efficacy of lopinavir/ritonavir in COVID-19 (22, 30–32). Consequently, the lopinavir/ritonavir combination was removed from the guidelines by SBMH and reserved only for pregnant patients, April 2, 2020 (10). In the current study, lopinavir/ritonavir did not show a beneficial association with parameters studied. In contrast, its administration was associated with longer hospital stays, though it was used in a small proportion of patients during the first few weeks of the pandemic. Although unadjusted analysis showed that it was related to increases in the risks of ICU admission and the need for IMV, additional analysis showed that this increase was not significant for either condition.

Favipiravir is a purine nucleic acid analog that inhibits RNA-dependent RNA polymerase. It is used in Japan as an anti-influenza drug and was also approved for the treatment of COVID-19 patients in China in March 2020 after Wang et al. showed its *in vitro* efficacy against SARS-CoV-2 (18, 20). However, the drug was not included in guidelines from the WHO or the CDC (13, 33). Favipiravir was implemented as a new recommendation for progressive mild, moderate and severe cases in the SBMH Treatment Guidelines on March 23, 2020, and has retained its standing as a treatment for COVID-19 since then (10). This drug was widely accepted and used by pulmonologists who had to deal with those moderate and severe COVID-19 cases in Turkey during these first months of pandemic (10). Since favipiravir is thought to reduce the duration of symptoms in patients (34), the recommendation of the Ministry of Health of Turkey regarding favipiravir is still ongoing (10). Although some physicians in Turkey are reluctant to prescribe favipiravir to patients, most of them recommend it per SBMH guidelines (10) to avoid legal problems and inform the patient about the effects and adverse effects of the drug.

Our study does not confirm the recommendation by SBMH guidelines, as favipiravir was not associated with any improvement in clinical parameters studied in our study population. These findings are, however, in agreement with those of others, who reported that clinical recovery of patients receiving favipiravir was no better than that of patients receiving lopinavir/ritonavir, with respect to the length of hospital stay, ICU admission or intubations (35). Similarly, in another multicenter, randomized, interventional study, there was no difference between favipiravir, and chloroquine with respect to the length of hospitalization or the need for mechanical ventilation (36). A recent meta-analysis of 12 clinical trials did not show a significant difference in fatality rate and mechanical ventilation requirement between favipiravir treatment and standard care in moderate and severe COVID-19 patients (37).

In our study, favipavir was associated with longer hospitalization and increased risk of ICU admission and IMV requirement in our study population. In line with these findings, a retrospective study on 824 COVID-19 patients reported that ICU admission rates were significantly higher in patients treated with favipiravir (38). However, in their study evaluating the ICU admission rates of COVID-19 patients in two different time periods, i.e., before and after the addition of favipiravir, Guner et al. found that the percentage of cases admitted to the ICU dropped, while the intubation rate among ICU patients decreased after favipiravir was implemented (39). Although authors hypothesized that favipiravir might have played a role in decreasing ICU admissions and intubation rates, they could not unequivocally propose either clinical use of favipiravir or change in treatment algorithms in favor of it (39).

Although favipiravir was reported to have a well-characterized safety profile (40), it has common adverse events including gastrointestinal side effects, increases in serum levels of ALT, AST, and uric acid (40, 41), and prolongation of QT interval in ECG (42, 43). Hepatic and renal side effects, as well as QTc prolongation, may be encountered during lopinavir/ritonavir use for COVID-19 (30, 44). In our study population, lopinavir/ritonavir was associated with increased risks of both hepatic and renal toxicity, and favipiravir and azithromycin were associated with increased risk of hepatotoxicity. None of our drugs was associated with increased risk of QTc prolongation. Although there was a weak association between favipiravir use and QTc prolongation, further analysis, which was adjusted for concomitant HCQ, azithromycin use, number of antibiotics, presence of hypertension or heart failure, revealed that it was not significant.

Our study was not designed to investigate the mechanisms underlying the side effects of drugs that were used in the study patients; however, a possible explanation for the worse morbidity outcomes in lopinavir and favipiravir regimens could be due to the severity of the disease (35). Because, in the beginning of the pandemic, favipiravir was administered to severe or mild cases, which were unresponsive to the initial treatment of HCQ according to the SBMH treatment guidelines (10). However, these drugs retained their association with negative outcome parameters such as longer hospital stay, ICU admission and need of IMV, respectively, after further analysis adjusting these for disease severity, number of comorbidities, age, sex, and use of drugs including antibiotics, HCQ, and azithromycin. Another explanation for negative impacts of these drugs could be due, at least in part, to their adverse effects such as hepatotoxicity and nephrotoxicity.

On October 22, 2020, the FDA approved another anti-viral drug, remdesivir for the treatment of COVID-19 (45) on studies suggesting its beneficiary effects (46–48). In a double-blind, randomized, placebo-controlled trial in adults with COVID-19, remdesivir was found to be effective comparing to placebo in shortening the time to recovery in adults who were hospitalized with COVID-19 and had evidence of lower respiratory tract infection (48). Later, clinical trials reported positive effects of another repurposed anti-viral compound, molnupiravir during the pandemic (49, 50). In a phase 3, double-blind, randomized, placebo-controlled trial of 1,433 patients, molnupiravir reduced the risk of hospitalization or death in the study population (49). In the light of such randomized studies, on December 23, 2021, the FDA issued an emergency use authorization (EUA) for molnupiravir for the treatment of mild to moderate COVID-19 in adults (50). However, these drugs were not available, when we conducted our study.

A comprehensive overview concerning several repurposed drugs and the newly approved vaccines, concluded that repurposed drugs could only be complementary to newly approved SARS-CoV-2 vaccines to attain overall mitigation of the COVID-19 pandemic (51). Although repurposing the existing drugs could be a strategy in such conditions, this strategy should not be applied without the reliable scientific evidence supporting their safety and efficacy. Our study and others have demonstrated that drugs repurposed based on their limited *in vitro* cellular (18) and clinical effects (19, 34), may not show their efficacy in the clinical setting. They may even have adverse effects on patients’ health. Therefore, even under pandemic conditions, it is clearly unacceptable that some treatments are applied to patients without clear scientific evidence, contrary to the principles of evidence-based treatment (52).

This study has some limitations due to its retrospective nature. TTD-TURCOVID 19 was a country wide registry; therefore, all pulmonology clinics with COVID 19 management facilities were invited to take part in the study, and 26 centers including university hospitals, large tertiary hospitals, secondary care hospitals and private hospitals joined the study. The standards for patient assessment, monitorization, number of pulmonologists, data records, and data collection were not uniform and same in all centers. Therefore, there were missing of data on treatment protocol of patients, drugs used, and location where the initial treatment started. Although most of the centers recorded the data on adverse effects of the drugs such as hepatotoxicity, nephrotoxicity and QTc prolongation, these were not available for some patients. It was not possible to do a head-to-head comparison of drugs used, since the study period comprised the beginning and early months of the pandemic when there were many unknowns about treatment options and the urgent need to treat patients. Therefore, drugs that were found to have limited evidence of antiviral or anti-inflammatory activity against COVID-19 were implemented, and most of the patients received a combination of drugs.

## Conclusion

Our study demonstrated that none of repurposed drugs for COVID-19 treatment had a positive association with clinical improvement. On contrary, favipiravir lopinavir/ritonavir and HCQ were associated with longer hospitalization, and additionally, favipiravir was significantly associated with increased the risk of ICU admission and the requirement for IMV suggesting that they can worsen the clinical outcomes of COVID-19 patients. Therefore, even under pandemic conditions, the principles of evidence-based treatment should not be dismissed, and effective pharmacologic agents with proven safety profile and efficacy should be applied.

## Data availability statement

The original contributions presented in this study are included in the article/Supplementary material, further inquiries can be directed to the corresponding author.

## Ethics statement

The studies involving human participants were reviewed and approved by Institutional Review Board of Gazi University Faculty of Medicine, Ankara, Turkey (356/22.05.2020). Written informed consent for participation was not required for this study in accordance with the national legislation and the institutional requirements.

## Author contributions

CB, NKk, HBa, OI, FO, AS, ASC, BD, and AK designed the study. CB, NKk, PC, SAB, OKa, PA, II, AAk, YS, OB, EA, SM, CC, AD, BK, BB, HO, GO, ZT, BE, VA, OKi, ME, TU, OAl, EN, OAt, AAy, DC, YG, FF, FD, NKs, MT, GG, SA, TeT, TiT, OO, OD, PY, IB, HBo, IO, SB, BG, FB, OE, IH, HK, GS, OU, MA, GA, BD, EC, EG, OG, YH, MO, and ASC collected the data. SK and SAN analyzed the data. CB, NKk, SK, and HBa searched the literature and wrote the manuscript. CB, NKk, SK, SAB, PC, and HBa edited and revised manuscript according to journal’s instructions. CB, NKk, SK, PC, SAB, and HBa edited and controlled the final version of the manuscript. All authors approved the final version of the manuscript.

## Supplementary material

Supplementary Figure 1 Distribution of patients with different drug combinations (Ose, Oseltamivir; HQC, Hydroxychloroquine; Azi, Azithromycin; Fav, Favipiravir; Lop/Ri, Lopinavir/Ritonavir).
