# Supplementary figures and images for: The association of antiviral drugs with COVID-19 morbidity: The retrospective analysis of a nationwide COVID-19 cohort
Source: Front Med (Lausanne). 2022 Aug 31;9:894126. doi: 10.3389/fmed.2022.894126 (PMC9471091; doi:10.3389/fmed.2022.894126)

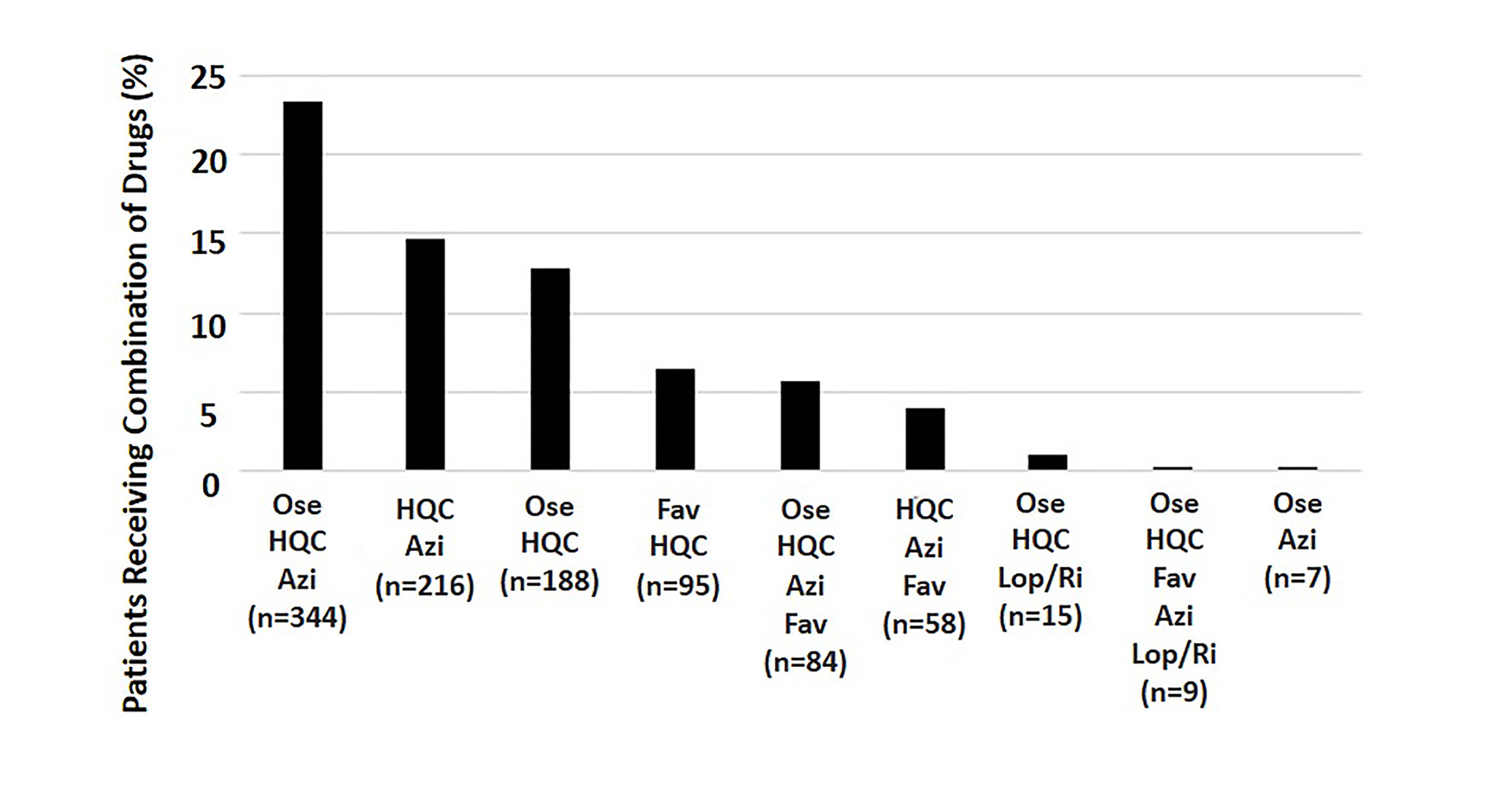

Supplement: Supplementary Figure 1 — Distribution of patients with different drug combinations (Ose, Oseltamivir; HQC, Hydroxychloroquine; Azi, Azithromycin; Fav, Favipiravir; Lop/Ri, Lopinavir/Ritonavir). [file Image_1.JPEG]
